# HuVarBase: A human variant database with comprehensive information at gene and protein levels

**DOI:** 10.1371/journal.pone.0210475

**Published:** 2019-01-31

**Authors:** Kaliappan Ganesan, A. Kulandaisamy, S. Binny Priya, M. Michael Gromiha

**Affiliations:** 1 Department of Biotechnology, Bhupat and Jyoti Mehta School of BioSciences, Indian Institute of Technology Madras, Chennai, Tamilnadu, India; 2 Advanced Computational Drug Discovery Unit (ACDD), Institute of Innovative Research, Tokyo Institute of Technology, Midori-ku, Yokohama, Kanagawa, Japan; Koç University, TURKEY

## Abstract

Human variant databases could be better exploited if the variant data available in multiple resources is integrated in a single comprehensive resource along with sequence and structural features. Such integration would improve the analyses of variants for disease prediction, prevention or treatment. The HuVarBase (HUmanVARiantdataBASE) assimilates publicly available human variant data at protein level and gene level into a comprehensive resource. Protein level data such as amino acid sequence, secondary structure of the mutant residue, domain, function, subcellular location and post-translational modification are integrated with gene level data such as gene name, chromosome number & genome position, DNA mutation, mutation type origin and rs ID number. Disease class has been added for the disease causing variants. The database is publicly available at https://www.iitm.ac.in/bioinfo/huvarbase. A total of 774,863 variant records, integrated in the HuVarBase, can be searched with options to display, visualize and download the results.

## Introduction

Human variant databases are being created frequently with specific scopes and contents. Their significance ranges from accurately predicting the disease [1] to achieving personalized medicine [2]. However, as the scopes of these databases differ, variant related data is inevitably spread across a number of databases such as 1000 Genomes [3], COSMIC [4], ClinVar [5], SwissVar [6], Humsavar (https://www.uniprot.org/docs/humsavar) etc. These databases have various limitations, including the fact that the data structures of these databases are not compatible with each other. As a result, obtaining comprehensive information on a variant of interest is still challenging for geneticists, biologists and clinicians [7]. Although integrating resources for the analysis of variant data obtained through Next Generation Sequencing (NGS) has been reported earlier [8], the integration is for tools and pipelines and not for the variant data.

Further, currently available variant databases do not include protein level data namely sequence, structural or functional information about the protein which has the variant. Moreover, for disease causing variants, the disease class information is not available in the current databases. Recently, Kulandaisamy et al. [9] reported the MutHTP, wherein, gene level and protein level information related to disease causing and neutral variants have been compiled in a comprehensive manner. However, MutHTP is limited to variants reported in human membrane proteins only.

We present here the HuVarBase (HUmanVARiant dataBASE), which is a comprehensive database for collating human variant data along with protein level data such as secondary structure of the residue in which the mutation has occurred, protein domain, subcellular localization, post-translational modification and function of the protein in which the variant is reported. In addition, if a variant leads to disease, then the disease class information also is included. The database has been implemented in a searchable server and made available online

## Materials and methods

### Datasets and curation

**Fig 1:**
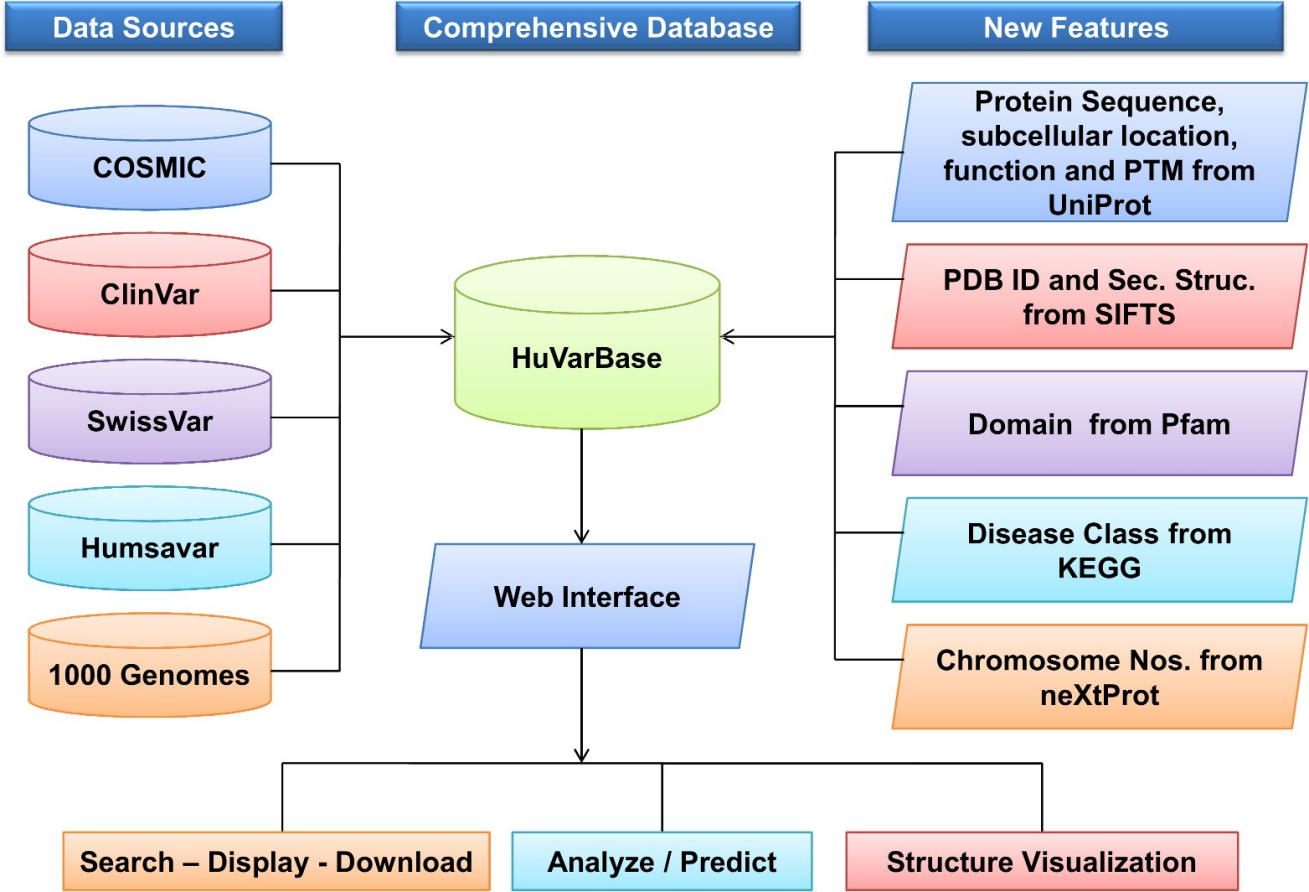
Schematic diagram describing the data collection, features and applications of HuVarBase.

An outline of the datasets and curation steps are given in Fig 1. The current human variant datasets available in 1000 Genomes [3], ClinVar [4], COSMIC [5], SwissVar [6] and Humsavar (https://www.uniprot.org/docs/humsavar) were downloaded and merged based on their UniProt [10] identifiers. If the UniProt identifiers were not available in the dataset, then the same were obtained from UniProt database using the Gene Name or sequence identifiers. For the COSMIC [5] dataset, variants reported in two or more tumor samples were designated as cancer causing (driver mutations) [11] and included in our databases. Remaining COSMIC variant data were not included in our database. If a variant is reported in more than one database, then the variant data is merged and the respective databases are mentioned in the source column. Protein sequences corresponding to the UniProt identifiers were obtained from the UniProt server. For a given variant, the neighboring residue information(three residues each, before and after the mutated residue) was taken from either the UniProt canonical protein sequence, or the UniProt isoform protein sequence, whichever had the amino acid residue in which the variant was reported. Protein Data Bank—PDB [12] identifiers of proteins and the secondary structure information of the variant residue were obtained from the SIFTS [13] database. Chromosome number and & genome position corresponding to the variants were collected from the neXtProt [14] database.

Subcellular localization, function and post-translational modifications of the proteins were obtained from the UniProt database. Protein domains in which the variant residues were present were obtained from the Pfam (https://pfam.xfam.org) database [15]. The disease classes were obtained from the KEGG [16] database based on the disease description. If the disease description does not match with that of the KEGG database, then other sources like Genetic and Rare Diseases Information Center (https://rarediseases.info.nih.gov/) [17] and Genetic Testing Registry (https://www.ncbi.nlm.nih.gov/gtr/) [18] were referred to obtain the name of the disease or synonyms of the disease. Then using that particular information, disease class was obtained from KEGG database. The present scope of the HuVarBase is to include small-scale variants of ‘missense’, ‘insertion’ and ‘deletion’ types along with the ‘non-sense’ type. Large-scale variations like large copy number variations will be added in future versions of HuVarBase.

### Web server and site

HuVarBase is available online at https://www.iitm.ac.in/bioinfo/huvarbase/ and it is meant to be used for non-clinical academic purposes only. The server works on a Linux-Apache-MySQL-PHP (LAMP) architecture. Most fields in this database are searchable by either entering keywords or by choosing a keyword in the drop down menu. Users can also choose the required fields to be shown in the search results. Hyperlinks are given in the search results to, GeneCards [19] for information on the gene in which the given variant has occurred, dbSNP server [20] for rs ID numbers, UniProt server for information on the protein of the variant, Jmol (http://www.jmol.org) to view the 3D structure of the protein with the variant residue, the protein sequence with color-coded residues to differentiate neutral and disease causing variants and to the source databases. The search results can also be downloaded as a single file. The Frequently Asked Questions section contains answers to common questions raised by the users. A brief tutorial is also available on how to search and obtain variant data from HuVarBase.

## Results

**Fig 2:**
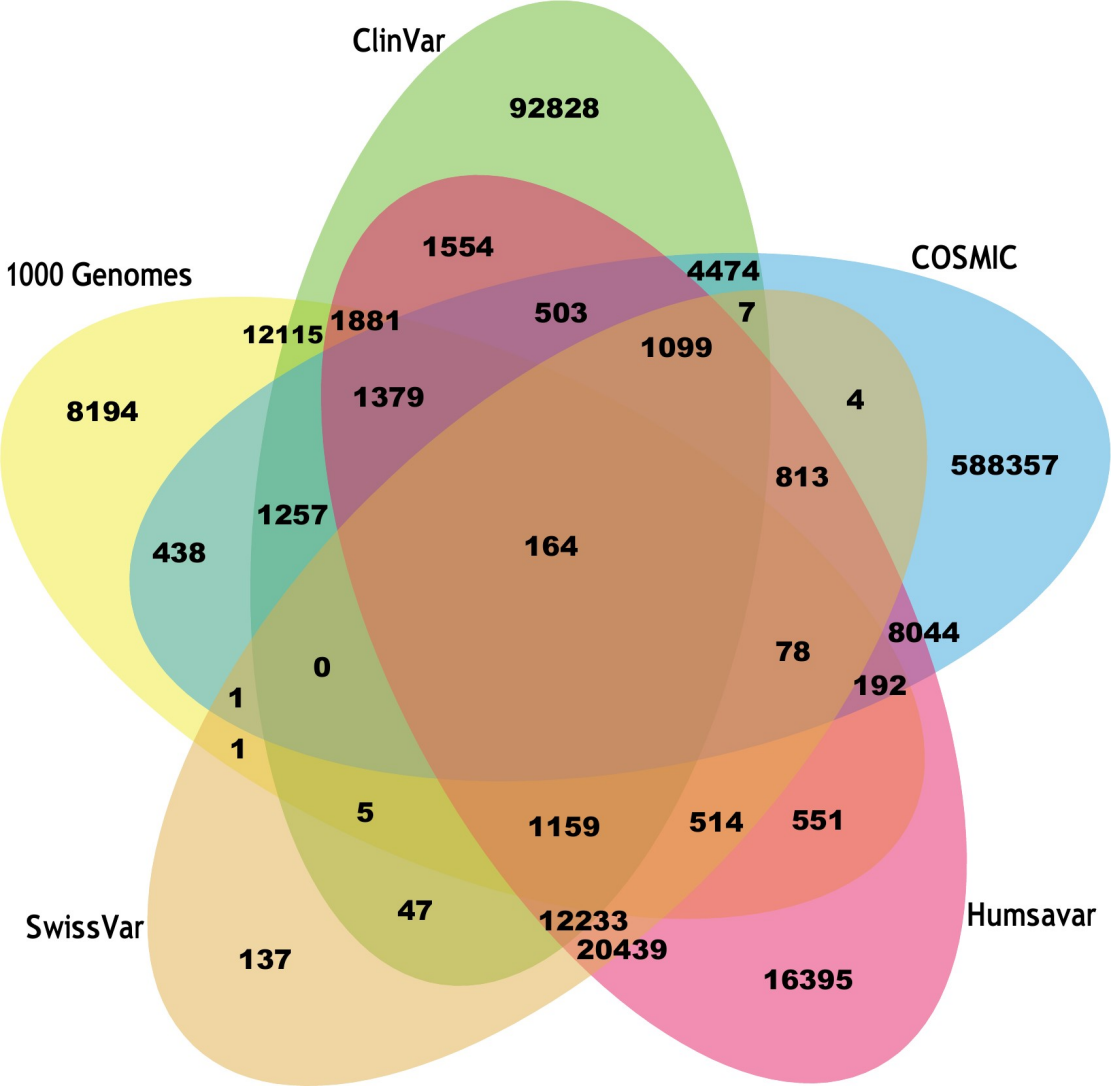
Venn diagram depicting the number of variants contributed from each of the data sources.

HuVarBase database includes 702,048 disease causing variants of which, 652,399 are ‘missense’, 10,174 are ‘nonsense’, 8,850 are ‘insertion’, and 30,625 are ‘deletion’ variants. There are 72,815 neutral variants (i.e. limited or no evidence on the pathogenic role of the mutation) of which 66,191 are ‘missense’, 2,885 are ‘nonsense’, 259 are ‘insertion’, and 3,480 are ‘deletion’ variants. In total, there are 774,863 variants reported from 18,318 proteins. The number of variants contributed from each of the data sources to the HuVarBase is depicted in Fig 2.

For a given variant record, the fields included are; i) Gene level data: name of the gene which has the variant, chromosomal coordinates, DNA mutation, type of the mutation, dbSNP [20] rs ID number if available and origin, ii) Protein level data: protein mutation, UniProt and PDB IDs of the corresponding protein, UniProt ID of the canonical or isoform of the protein in which the mutation has been reported, neighboring amino acids of the mutated amino acid, secondary structure of the particular amino acid residue, conservation score of the residue change, function of the protein, protein domain in which the mutation has occurred, subcellular localization of the protein and post-translational modifications of the protein, and iii) others: Disease or tissue type in which the variant has been reported, disease class and the source database. Statistics regarding the database such as disease class frequency matrices for the disease causing and neutral mutations, etc. are given in the web server. Table 1 gives a comparison of features included in currently available human variant databases with HuVarBase.

**Table 1:** Comparison of features in HuVarBase with existing databases.

Features	Humsavar	SwissVar	1000 Genomes	COSMIC	ClinVar	MutHTP	HuVarBase
Gene name	Yes	Yes	Yes	Partial	Yes	Yes	Yes
Chromosome number	No	No	Yes	Yes	Yes	Yes	Yes
Origin of mutation	No	Yes	No	Yes	Yes	Yes	Yes
DNA Mutation	No	No	No	Yes	Yes	Yes	Yes
Type of mutation	Missense	Missense	Missense	All	All	Missense, Insertion, Deletion	Missense, Nonsense, Insertion, Deletion
rs ID number	Yes	Yes	Yes	No	Yes	No	Yes
UniProt ID	Yes	Yes	Yes	Partial	Partial	Yes	Yes
3D structure (PDB)	No	Yes	No	Yes	No	Yes	Yes
Disease class	No	No	No	Yes	No	Yes	Yes
Conservation score	No	No	No	No	No	Yes	Yes
Neighbouring residues	No	No	No	No	No	Yes	Yes
UniProt ID of Isoforms	No	No	Yes	No	No	Yes	Yes
Protein Domain	No	No	No	No	No	Yes	Yes
Protein Function	No	No	No	No	No	No	Yes
Subcellular Location	No	No	No	No	No	No	Yes
PTM*	No	No	No	No	No	No	Yes
Secondary structure	No	No	No	No	No	No	Yes
Organism	Human	Human	Human	Human	Human	Human (membrane proteins)	Human

* PTM—Post Translational Modifications

## Applications

The comprehensive HuVarBase facilitates searching for a variant and obtaining variant-related sequence and structural information for viewing or downloading. The HuVarBase includes small scale variant types such as ‘missense’, ‘insertion’and ‘deletion’ along with the type ‘non-sense’ in the human genome. The applications of HuVarBase range from analysis, disease prediction to personalized medicine as exemplified by earlier efforts [21–23]. Analysis of the variants can be performed at sequence and structural level, in order to understand the effects of mutations leading to disease. Prediction algorithms requiring a comprehensive variant dataset can make use of the vast dataset available with the HuVarBase. The database will be updated periodically. The updates will be on a quarterly basisand the update information will be reflected in the ‘What’s New’ section of the web server.
